# Perceived Self-Efficacy and Coping Styles Related to Stressful Critical Life Events

**DOI:** 10.1371/journal.pone.0067571

**Published:** 2013-07-12

**Authors:** Simonetta D'Amico, Assunta Marano, Maria Angela Geraci, Emanuele Legge

**Affiliations:** 1 Department of Life, Health and Environmental Sciences, University of L'Aquila, Coppito (AQ), Italy; 2 Independent Practice in Psychotherapy, L'Aquila, Italy; 3 Department of Mental Health, Local Health Agency of the National Health System, L'Aquila, Italy; Federal University of Rio de Janeiro, Brazil

## Abstract

**Objective:**

The study examined the personal resources, Self-efficacy and Coping Strategies, in a sample of pre-adolescents who experienced an emotionally and socially critical event, such as the earthquake of the 6^th^ of April 2009, related to age and gender.

**Methods:**

198 pre-adolescents, 84 girls and 114 boys (Age Mean 12 years), attending Secondary School in L'Aquila meso-seismic area. The emotional, behavioural and social capacities have been assessed with specific questionnaires administered collectively ten months after the earthquake.

**Results:**

Multidimensional analyses produced differentiated profiles according to gender and age: self-efficacy-perception and coping strategy profiles, based on quartiles calculations, revealed the difficulties of the subjects in estimating their ability to cope with the world of relations and emotions after the critical event.

**Conclusions:**

The intervention could be specific to the cognitive, emotional and relational state of children and adolescents and differentiated before (prevention), during (intervention) and after the event (intervention and prevention).

## Introduction

Perceived self-efficacy, related to different domains of functioning in which human experience occurs, contributes towards building the psychological effectiveness and psychosocial adjustment-styles of individuals in relation to their particular environments [1], [2].

Conviction of perceived self-efficacy defined as “people's beliefs about their capabilities to exercise control over events that affect their lives” [3], sustain and regulate cognitive, motivational and affective processes and impact upon adjustments of the self in such a way as to favour coping of managing the demands of everyday life [4], [5].

The literature on developmental determinants and mechanisms of social adaptation of well-being and health-promotion [6], [7], [8], [9] shows that those who believe they can deal effectively with potential stressors, face and handle stress better, adopting more efficacious coping styles “but if they believe they cannot control aversive events they distress themselves and impair their level of functioning” [3]. In this view the regulation of the behaviour of an individual regarding objective choices and actions, is strongly influenced by belief in their ability to increase their motivation level, to draw on cognitive resources and carry out the actions to exercise control.

The social cognition theory posits the existence of an agency model of adaptation and change which enables individuals to play a proactive role in adjustment processes, instead of simply submitting experiences where environmental stress factors impact upon features of personal vulnerability. It states that people are self-organising, proactive, self-regulating, and self-reflecting. Therefore, they are contributors to their life circumstances not just products of them.

According to this theory, even in highly problematic conditions, individuals will select needs and exigencies from the context capable of creating developmental occasions in keeping with their goals and values: while meeting the demands of social pressures and satisfying their own physical and psychic needs, individuals actively influence both systems [4]. This way, those with high self-efficacy levels tend to consider the difficulties and obstacles with less apprehension and, in some cases, even see them as occasions to put themselves to the test, confident as they are of being capable of coping with them efficaciously, unlike, those with low self-efficacy levels who would be more likely to feel themselves in danger, exacerbating adversity and underestimating potential and opportunity [10], [11].

States of uncertainty regarding important issues throw people into a state of confusion: those who are capable of modifying the course of events significantly are equally capable of foreseeing them. Foreseeability favour adjustment.

In cases of natural disasters, like earthquakes, the destructive force of the events affects entire communities; not only by their dimension/gravity but also by the sudden changes to habitual individual and collective living conditions they inevitably cause.

In situations like this, children and adolescents are, in many ways, more vulnerable because of the developmental phase they are going through where emotional, affective and cognitive systems are still evolving.

In the case of very young children, for example, whose crucial reference appears to be the adult, the attachment system may be undermined, creating a sense of insecurity; in the case of older children, capable of reading the event cognitively, affective and relational forecasting may be affected and make it difficult for them to envisage their future.

The difficulties encountered when trying to read “unexpected” and “unforeseeable” events and the uncertainty that follows, can take the form of several kinds of social-adjustment difficulties including psychopathological disorders.

The specialist literature which has investigated the psychological consequences of natural disasters such as earthquakes, floods and hurricanes, reports traumatic and distressed reactions on the part of the majority of survivors [12], [13] listing a series of disorders caused by the events, along a continuum, including post-traumatic stress disorder (PTSD), major depressive disorder (MDD), alcohol abuse, anxiety and somatisation disorders, behavioural problems and a number of other psychic ailments, performance and psychological reactivity disorders, reactions which can seriously compromise the intrapersonal, interpersonal and occupational functioning of survivors.

The seismic event of the 6^th^ of April 2009, which had disastrous consequences for the city of L'Aquila and its surroundings (6.3 on the Mw (moment magnitude) scale; 5.8 on the local magnitude Richter; Io  =  IX MCS (macro seismic impact) on the Mercalli-Cancani-Sieberg scale), exposed the entire population to terror and tension both at the moment of crisis and for months afterwards.

Children and adolescents from the L'Aquila area had to cope with critical adjustment to a “new normality” requiring considerable reorganisation of their life styles, cycles and prospects.

In these conditions, a research-action project, aimed at preventing and treating disorder symptoms, was set up. It aimed at evaluating the personal resources (cognitive-affective self-efficacy and coping strategies) of the children and adolescents in question by creating preventive action solutions, such as psychological consultancies, individual and group counselling units. The final goal of this research-action initiative was to favour conscious and lasting changes of a three-fold nature: cultural, psychological, socio-political.

Present research is part of broader investigation aimed at identifying impact-factors in traumatic conditions in relation to the gravity of the experience and aims at describing the emotional, behavioural and social capacities of a group of pre-adolescents who experienced an emotionally and socially critical event, such as the earthquake of the 6^th^ of April 2009 undoubtedly was.

## Methods

The study protocol was approved by the local Institutional Review Board and was conducted in accordance with the Declaration of Helsinki with the written consent signed by the parents of each research participant.

### Participants and Setting

The sample group was composed of 198 pre-adolescents, 84 girls and 114 boys (average age 12 years, 6 months), who experienced a severe traumatic event, the earthquake of the 6^th^ of April 2009 (Table1). All the boys and girls belonging to this group attended Junior Secondary School (JSS) within the meso-seismic area of L'Aquila (The National Institute for Geophysics and Volcanology, INGV).

**Table 1:** Sample group's demographical characteristics: numbers and average ages of the subjects according to the classes attended and gender.

Class Attended	Gender	N	Average Age
I JSS Class	Males	48	11 years, 4 months
	Females	23	11 years and 6 months
II JSS Class	Males	31	12 years and 7 months
	Females	29	12 years and 6 months
III JSS Class	Males	35	13 years and 6 months
	Females	32	13 years and 6 months

The data were gathered ten months after the earthquake. The assessment was carried out availability of questionnaires administered collectively in the new temporary school complex (MUSP) attended by the young people in question. The inclusion criteria was the presence in the meso-seismic area of L'Aquila during the night of the earthquake: we collected demographic data & exposure to L'Aquila earthquake information. The sample group did not include subjects whom the psycho-pathological clinic reported as suffering from post-traumatic stress disorder (PTSD).

### Measures

To assess the emotional, behavioural and social capacities of the subjects, a series of scales whose psychometric value and content features are described here, were used:

The subjects' Social Self-efficacy [14] was measured by availability of a scale of 13 items, with five answer options, aimed at evaluating the subjects' convictions of their own ability to form and maintain social relations, assert their own opinions, cope with different kinds of interpersonal conflict. The Cronbach reliability coefficient was .86 (Example of items: “How capable are you of…”: “expressing your own opinion when, in the company of your friends, you are discussing something”, “working in a team”).

The subjects' Regulatory Scale of Perceived Self-efficacy [14] was measured by availability of a scale of 12 items with five answer options, aimed at appraising the convictions the girls and boys had regarding their ability to resist peer pressure seeking to involve them in risky action (smoking, drinking, committing a traffic offence, etc.).

The subjects' Scale of Perceived Self-efficacy in Coping with Negative Emotions and the Scale of Perceived Self-efficacy associated with Expression of Positive Emotions [15]:

- Perceived Self-efficacy associated with Expression of Positive Emotions (i.e. happiness, enthusiasm, tenderness, affection or contentment), measured by availability of 7 items. The Cronbach reliability coefficient was .83 (Example of items: “How capable are you of…”: “expressing your happiness when something pleasant happens to you”, “demonstrating your satisfaction when you reach goals you set for yourself”).
- Perceived Self-efficacy in Coping with Negative Emotions (i.e. dejection, frustration, bad mood, discontent or anger), by availability of a scale of 8 items. Cronbach reliability coefficient was .83 (Example of items: “How capable are you of …”: “becoming discouraged by adversity”, “remaining calm in stressful situations”).

Il Brief-Cope Coping Orientation towards Problems Experienced – new Italian version of C.O.P.E. (NVI) [16], [17] devised to measure coping strategies on five levels: problem-oriented coping-strategies (pos); avoidance strategies (as), social-support strategies (sss); positive attitude (pa); transcendent-oriented (to). On the basis of the construction criteria of the original version (60 items) and the characteristics of internal homogeneity of the five (5) scales the adaptation for adolescents contains 28 items.

#### Overview of Statistical Analyse

Descriptive analyses were carried out, estimating average tendency and dispersion measurements and quartiles for the self-efficiency and coping-strategy scales, calculated in relation to normative reference values (25^th^, median and 75^th^). In the case of the multidimensional analyses, univariate (ANOVA) multivariate (MANOVA) statistical-calculation methods were applied; we used a Bonferroni adjusted alpha level of .05.The independent variables were gender (2 levels: M e F) and class attended at school (3 levels: I, II and III year of JSS) while degrees of self-efficacy and coping strategies were the dependent variables.

## Results

### Conviction of efficacy

Table 2 contains average values and standard-scale deviations for Social Self-efficacy (*Social*), Regulatory Perceived Self-efficacy (*Regulation of emotion*), Perceived Self-efficacy in Coping with Negative Emotions (*Negative emotion*) and Perceived Self-efficacy associated with Expression of Positive Emotions (*Positive emotion*), with separate data for gender (male/female) per group examined (I, II and III year JSS).

**Table 2:** Average (M) and standard deviation (SD) values for Conviction of self-efficacy calculated separately on the basis of gender (males/females) for the three groups examined (I, II and III year of JSS).

JSS	Indicators	Social		Regulation of Emotion		Positive Emotion		Negative Emotion	
		*M*	*F*	*M*	*F*	*M*	*F*	*M*	*F*
I	M	4,39	4,15	1,60	1,72	3,73	3,69	3,20	2,97
	*DS*	*0,56*	*0,55*	*0,34*	*0,33*	*0,42*	*0,45*	*0,79*	*0,62*
II	M	4,20	4,28	1,64	1,51	3,80	3,94	3,24	2,90
	*DS*	*0,69*	*0,68*	*0,40*	*0,49*	*0,46*	*0,42*	*0,74*	*0,65*
III	M	4,33	4,21	1,62	1,74	3,59	3,71	3,34	3,12
	*DS*	*0,48*	*0,58*	*0,34*	*0,29*	*0,44*	*0,48*	*0,55*	*0,63*

From the descriptive analyses it emerges that the younger boys (I) had a higher perception of Social Self-efficacy compared to the girls (Table 2) while the older girls (III) declared being more enterprising and capable of maintaining social relations compared with males of the same age. Furthermore, they claimed to be more assertive when upholding their opinions (Regulatory Perceived Self-efficacy scale).

Also in the case of the degree of perception the young people examined had of their ability to resist peer pressure seeking to involve them in risky action (smoking, drinking, committing a traffic offence, etc.) the value was higher for the older girls than for the boys of the same age (Regulatory Perceived Self-efficacy scale).

Finally, as to perceived self-efficacy when coping with emotions, (Perceived Self-efficacy in Coping with Negative Emotions) the boys (I, II and III) seemed to be better able to cope with negative emotions while they were less able to cope with expressing positive emotions (Perceived Self-efficacy associated with Expression of Positive Emotions) revealing a decidedly separate trend for males and females in all three groups considered (I, II and III year JSS).

The variance analyses (Manova and Anova) for the adaptation-level evaluation indicators revealed different profiles for the gender variable only with regard to negative emotions (F = 4,87; p = .02) while the self-efficacy scales (social and regulatory) yielded statistically significant results availability of the MANOVA (2×3×3) factor model.

**Figure 1:**
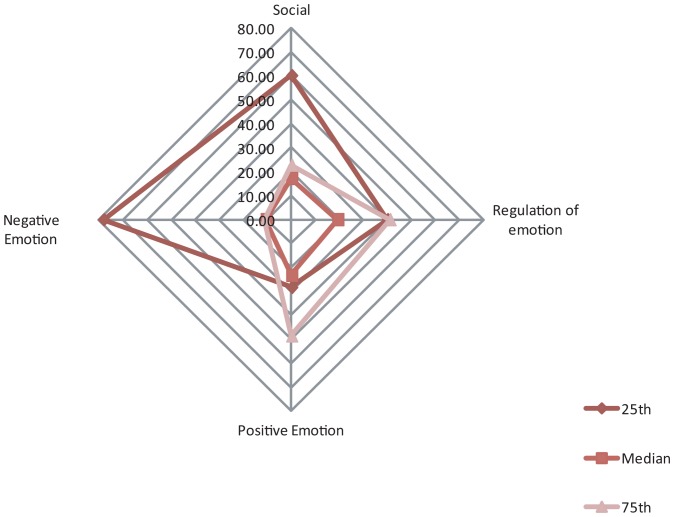
Radar graph Overall percentages of the conviction of self-efficacy values per quartile (25^th^, median and 75^th^).

To provide a more detailed reading of these data, the sample-group's distribution values, in terms of percentages of degrees of perception of self-efficacy for the three kinds of adequacy declared by the subjects in relation to the various self-efficacy categories tested (Figure 1), are described here. The quartile values (25^th^, median and 75^th^) were obtained from the normative data expressed in percentiles of the corresponding scales [15].

On the whole sample (Figure1), it appears that our subjects' self-perception of Social and Regulatory Self-efficacy is considerably “low”; 50% of them, in fact, declare to have a low sense of Social Self-efficacy (60.1%) and of Perceived Self-efficacy in Coping with Negative Emotions (78.3%).

The data regarding their ability to resist peer pressure seeking to involve them in risky action (smoking, drinking, committing a traffic offence, etc.) contains two practically equal but diametrically opposite groups: 40% declare to have a low level of conviction concerning their ability to resist, while 41% are convinced that they are perfectly able to cope with the issue.

Table 3 presents the sample-group distribution according to a categorised efficacy-evaluation profile based on perception-of-self-efficacy percentages by quartiles (25^th^, median and 75^th^) calculated separately according to gender (males/females) for the three groups examined (I, II and III year of JSS).

**Table 3:** Percentages of self-efficacy conviction by quartiles (25^th^, median and 75^th^) calculated separately according to gender (males/females) for the three groups examined (I, II and III year of JSS).

JSS	Indicators	Social		Regulation of Emotion		Positive Emotion		Negative Emotion	
		*M*	*F*	*M*	*F*	*M*	*F*	*M*	*F*
I	25^th^	58,33	69,57	47,92	26,09	27,08	30,43	72,92	82,61
	Median	12,50	17,39	18,75	30,43	16,67	30,43	12,50	13,04
	75^th^	29,17	13,04	33,33	43,48	56,25	39,13	14,58	4,35
II	25^th^	54,84	55,17	35,48	44,83	29,03	17,24	77,42	86,21
	Median	25,81	20,69	12,90	17,24	9,68	20,69	6,45	6,90
	75^th^	19,35	24,14	51,61	37,93	61,29	62,07	16,13	6,90
III	25^th^	60,00	65,63	51,43	25,00	28,57	37,50	77,14	78,13
	Median	14,29	15,63	14,29	25,00	37,14	28,13	8,57	15,63
	75^th^	25,71	18,75	34,29	50,00	34,29	34,38	14,29	6,25

This permits us to observe how different resistance-to-peer-pressure values are for males and females; only a quarter of the older girls stated finding it hard to regulate their emotions while over 50% of the boys admitted having greater difficulties on the same issue.

At the same time, it has been possible to detect how many of the young people claimed being less competent when it came to social relations or in coping with their emotions (I quartile, 25th) and how many felt more confident about their behaviour and/or their ability to regulate their emotions (III quartile, 75th).

### Coping strategies

For the coping-strategy scale too, the average values per answer were calculated. Table 4 contains average values and standard-scale deviations for coping-strategies (*Problem oriented*, *Avoidance strategies*, *Social support*, *Positive action*, *Transcendent oriented*) by gender (males/females) for the three groups examined (I, II and III year of JSS).

**Table 4:** Average (M) and standard deviation (SD) for Coping strategies calculated separately on the basis of gender (males/females) for the three groups examined (I, II and III year of JSS).

JSS	Indicators	Social Support		Avoidance		Positive Attitude		Problem-oriented		Transcendent-oriented	
		*M*	*F*	*M*	*F*	*M*	*F*	*M*	*F*	*M*	*F*
I	M	2,61	2,47	2,43	2,20	2,86	2,74	2,27	2,07	2,09	2,16
	*SD*	*0,44*	*0,72*	*0,43*	*0,37*	*0,46*	*0,50*	*0,48*	*0,34*	*0,53*	*0,73*
II	M	2,42	2,66	2,29	2,16	2,70	2,83	2,26	2,31	2,07	2,09
	*SD*	*0,65*	*0,69*	*0,37*	*0,45*	*0,52*	*0,59*	*0,37*	*0,43*	*0,60*	*0,54*
II	M	2,09	2,53	2,12	2,00	2,66	2,49	1,91	1,91	1,71	1,52
	*SD*	*0,35*	*0,58*	*0,52*	*0,57*	*0,50*	*0,55*	*0,25*	*0,38*	*0,43*	*0,31*

The sample group availed of all the coping subscales (Table 4) and of all the five subscales provided, the most frequently availed of by the three groups examined (I, II and III year of JSS) was to seek comprehension, information and venting emotions (social support coping). The only exception was that of the older girls (III JSS: M = 2.53; DS = 0.58) who claimed availability mostly of behaviour aimed at facing stressful conditions grounded in planning and acting (problem-oriented coping).

The variance analyses (Manova and Anova) of the strategies revealed the simple impact of the age variable. Denial and avoidance, recourse to drugs and medication, mental and behavioural detachment are the profiles that emerge on the basis of gender and age differentials. The use of avoidance strategies makes a statistically significant distinction (Tukey HSD p<.01) between the group of older boys and girls (average III JSS  = 14.43) and the younger ones (average I JSS  = 16.52) and between girls (average  = 14.77) and boys (average  = 16.08) yielding a significant result for the simple impact of the class-attended variable (F_(2, 195)_  = 7.07, p<.01) and for that of the gender variable (t_(195)_  = 2.76, p<.01).

As to the remaining coping styles (positive attitude, problem-oriented coping, social support tendency to transcend) the differences occurred only on the basis of the age variable.

Acceptance, containment and positive reinterpretation produced significant results (Tukey HSD p<.01) as regards the behaviour of the younger subjects (average I JSS  = 16.94) compared to that of the older ones (average III JSS = 15.46) yielding a significant result for the class-attended variable in relation to positive attitude (F_(2, 195)_  = 4.22, p<.05).

Likewise, problem-oriented attitudes grounded in planning and action characterised, in a statistically significant manner (Tukey HDS p<.01), the behaviour of the younger subjects (average I JSS = 13.24 ; average II JSS = 13.70) compared to the older ones (average III JSS  = 11.48). The simple effect of the class-attended variable in relation to the problem-oriented coping style yielded significant results (F_(2, 195)_  = 16.38, p<.05).

Similarly, the Social support coping style produced a significant result on the basis of the simple effect of the class-attended variable (F_(2, 195)_  = 4.26, p<.01). The class-attended variable permitting the discriminatory division of the sample group according to the item pool referred to seeking comprehension, information and venting emotion. In particular, the post hoc analyses revealed that the scale distinguishes, in a statistically significant manner (Tukey HDS p<.01), between subjects attending the first-year of JSS (average  = 15.38) and those attending the third-year class (average  = 13.80).

Finally, the stress-situation problem-oriented behaviour strategy based on religious sentiment and lack of a sense of humour distinguishes, in a statistically significant manner (Tukey HDS p<.01), between the younger (average I JSS = 8.45; average II JSS = 8.31) and the older subjects (average III JSS = 6.46). The simple effect of the class-attended variable was significant also in the case of the transcendent-oriented coping style (F_(2, 195)_  = 18.52, p<.01).

**Figure 2:**
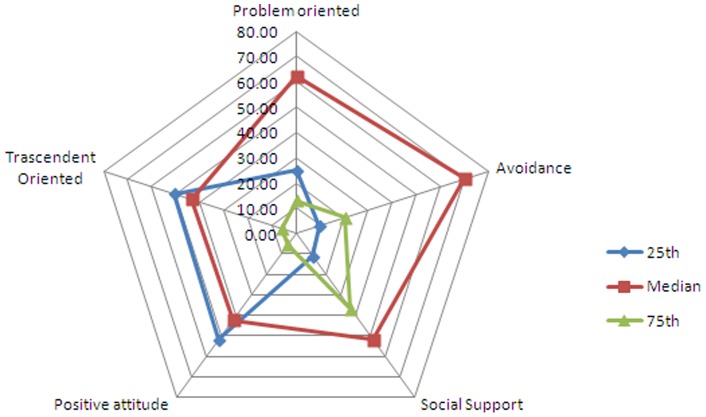
Radar graph Overall percentages for Coping strategies by quartiles (25th, median and 75th).

In an attempt to observe the flexibility parameter related to use of coping strategies (tab.5: 25^th^, median and 75^th^), and the coping strategies themselves, quartiles were calculated on the basis of the distribution data for the sample group. On the whole sample (Figure 2), over 50% of the subjects reported availability of strategies including avoidance, comprehension and venting emotions (social support).

The boys and the girls declared finding it difficult to adopt problem-oriented strategies involving religious sentiment and lack of a sense of humour (transcendence oriented) as well as acceptance, containment and positive reinterpretation of the event undergone (positive attitude).

Table 5 provides a more detailed reading of coping-strategy percentages by quartiles (25^th^, median and 75^th^) calculated separately according to gender (males/females) for the three groups examined (I, II and III year of JSS).

**Table 5:** Coping–strategy percentages by quartiles (25^th^, median and 75^th^) calculated separately by gender (males/females) for the three groups examined (I, II and III year of JSS).

				Avoidance		Positive Attitude		Problem-oriented		Transcendent-oriented	
		*M*	*F*	*M*	*F*	*M*	*F*	*M*	*F*	*M*	*F*
I	25^th^	8,33	27,27	2,08	4,35	4,17	13,04	37,50	47,83	37,50	34,78
	Median	75,00	68,18	64,58	82,61	47,92	52,17	47,92	52,17	54,17	47,83
	75^th^	16,67	4,55	33,33	13,04	47,92	34,78	14,58	0,00	8,33	17,39
II	25^th^	27,59	18,52	0,00	13,79	12,90	13,79	35,48	44,83	38,71	41,38
	Median	62,07	59,26	80,65	65,52	45,16	41,38	61,29	44,83	51,61	55,17
	75^th^	10,34	22,22	19,35	20,69	41,94	44,83	3,23	10,34	9,68	3,45
III	25^th^	48,57	25,00	20,00	18,75	8,57	18,75	77,14	71,88	65,71	84,38
	Median	51,43	53,13	65,71	68,75	65,71	59,38	22,86	28,13	34,29	15,63
	75^th^	0,00	21,88	14,29	12,50	25,71	21,88	0,00	0,00	0,00	0,00

This way, it was possible to observe the levels of adaptation to the situation in terms of a more or less rigid use of strategies [18]. Table 5 permits us to observe the difficulties encountered by the older boys and girls when endeavouring to pursue problem-oriented strategies based on religious sentiment and lack of humour (Transcendent-oriented M and F 75th = 0%) as well as problem-oriented strategies centred on planning and action (problem-oriented M and F 75th = 0%): about half of the subjects used all of the strategies (median value) except for the older boys and girls (III JSS). This datum underlines the importance of the developmental state of the emotional, affective and cognitive systems of subjects going through this particular life phase, a period requiring considerably greater recourse to cognitive elaboration of the reality.

## Discussion

The way in which children and adolescents understand and recall stressful life events like an earthquake, varies greatly according to age, and impact on emotional, behavioural and social capacities, as well as on overall state of health [19].

The impact of such a highly-charged emotional occurrence produces in developing subjects, a series of reactions related to the developmental phase the child or adolescent is going through, to the upheaval of everyday life, distress and the activation of adult carers, the destruction of what has been familiar, including homes and physical and social community structures [20], [21], [22].

Emotional numbing, flashbacks, insomnia and digestive and cognitive disorders are among the usual reactions to a traumatic event; active coping strategies are cognitive and behavioural responses designed to terminate exposure to stressors and help the individuals resume “normal living,” actively challenging themselves to engage in “doing something” to change how they consider traumatic experiences [23].

The consequences of a traumatic event of similar proportions require a renegotiation of the self and a reinterpretation of life in order to correctly reposition them in relation to new contexts and resources.

In other words, traumatic events impact upon the construction and stabilisation of the self-system producing a less efficacious adjustment to life contexts, even to the point of the most nefarious of psychopathological outcomes.

The focus on adjustment/maladjustment permitted us to grasp how profoundly the self-system had been influenced by the event itself, for example, when evaluating the subjects' judgement of their ability to resist peer pressure seeking to involve them in risky action (smoking, drinking, committing a traffic offence, etc.) and their perception of their ability to create and maintain social relations, to assert their own opinions, and cope with different kinds of interpersonal conflict.

The analyses we carried out produced differentiated profiles according to gender and age. The younger boys claimed a higher level of Social Self-efficacy compared to the girls while the older girls (II JSS) stated they were more enterprising and capable of maintaining social relations than their male peers, as well as being more assertive about their opinions.

A more detailed, in-depth reading of the self-efficacy-perception profiles, based on calculations of quartiles, revealed the difficulties the subjects encountered when estimating their ability to cope with the world of relations and emotions; over 50% of the sample group claimed being convinced of their Perceived Social Self-efficacy (60.1%) and of their Perceived Self-efficacy in Coping with Negative Emotions (78.3%). As to gender, only one quarter of the older girls from the sample group declared finding it hard to regulate their emotions.

The data regarding resistance of peer-pressure seeking to cause risky behaviour (smoking, drinking, committing a traffic offence, etc.) produce two numerically equal, though diametrically opposite groups; one with a low, the other with a high level of perceived efficacy.

A datum of this kind is alarming because the weakening of perception of the self-efficacy system reduces the system's ability to strengthen itself in order to oppose the causes of stress and influence the pursuit of effective resources to cope with situations perceived as threatening [24].

In the case of the use of avoidance strategies too, we came up with differentiated profiles, but in this instance differences regarded the subjects' state of development only.

Over 50% of the sample-group subjects claimed activating avoidance strategies (72.7%), seeking comprehension and venting emotion (social support). They used all the coping-strategy subscales (Table 4). Of the five subscales, the most commonly availed of by the three groups examined (I, II and III year of JSS) was seeking comprehension, information and venting emotions (social-support coping), except for the older girls who claimed adopting problem-oriented strategies based on planning and action (problem-oriented).

The difficulties encountered by the older boys and girls regards to recourse to problem-oriented solutions based on religious belief and lack of humour (transcendent orienting) as well as behaviour aimed at facing stressful situations characterised by planning and action (problem orienting) underlines the developmental state of their emotional, affective and cognitive systems at this particular stage of their lives, when considerably greater recourse to cognitive elaboration of the reality is required.

Despite the limitation, our investigation represents a step forward in the study of the personal resources, Self-efficacy and Coping Strategies, in a sample of pre-adolescents who experienced an emotionally and socially critical event, and suggest that future action needs to be brought to bear on the developmental aspects associated with hardship and the resources as emerging from the age-difference responses of the subjects examined here.

Future studies should examine the relation between individual variables and contextual variables such as social support and cooperativeness. Moreover, within the perspective of the study of developmental psychology becomes crucial to adopt a longitudinal perspective in order to verify the developmental trajectories and the adaptation to critical event.
